# Short-term impact of diurnal temperature range on cardiovascular diseases mortality in residents in northeast China

**DOI:** 10.1038/s41598-023-38129-2

**Published:** 2023-07-07

**Authors:** Xuan Kai, Zhimin Hong, Yang Hong, Xiaolei Wang, Chunyang Li

**Affiliations:** 1 Department of Mathematics, School of Sciences, Inner Mongolia University of Technology, Hohhot, 010051 China; 2 Department of Mechanics, School of Sciences, Inner Mongolia University of Technology, Hohhot, 010051 China; 3 Department of Ultrasound, Affiliated Hospital of Inner Mongolia Medical University, Hohhot, 010050 China; 4 Department of Neurology, Affiliated Hospital of Inner Mongolia Medical University, Hohhot, 010050 China

**Keywords:** Cardiovascular diseases, Statistics

## Abstract

It has been reported that cardiovascular disease (CVD) has become one of the major threats to global public health and is associated with climate change. Several previous studies have shown the influence of ambient temperature on CVD, but lack some evidence for the short-term effect of diurnal temperature range (DTR) on CVD mortality in northeast China. This is the first study to assess the correlation between DTR and CVD mortality in Hulunbuir located in northeast China. Daily CVD mortality data and meteorological data were collected from 2014 to 2020. A quasi-Poisson generalized linear regression with a distributed lag non-linear model (DLNM) was applied to exploring the short-term impact of DTR on CVD mortality. Stratified analyses by gender, age, and season were conducted and the short-term impacts of extremely high DTR on CVD mortality were investigated. In this study, a total of 21,067 CVD mortality cases were recorded in Hulunbuir, China from 2014 to 2020. Compared to the reference value (11.20 $^{\circ }$C, 50${\text{ th }}$ percentile), a “U-shaped” non-linear relationship between DTR and CVD mortality was observed, and extremely high DTR increased the risk of CVD mortality. The short-term effect of extremely high DTR occurred immediately and lasted up to 6 days. In addition, the male and the age $\ge$ 65 groups were more likely to be affected by extremely high DTR compared with the female and the age < 65 groups, respectively. The results also showed that extremely high DTR in cold season had a more adverse effect on CVD mortality than warm season. This study suggests that extremely high DTR for cold season should be paid enough attention to for residents in northeast China. The male and the age $\ge$ 65 groups were more vulnerable to the impacts of DTR. The study results may provide some suggestions for decision-making by local public health authorities to avoid the adverse impacts of high DTR, and improve the health of residents, especially vulnerable groups in cold season.

According to report, cardiovascular disease (CVD) has been one of the major threats to global public health, mostly affects middle-aged and older people^1–3^. In recent years, due to China’s rapid socioeconomic development and changes in living habits, the morbidity and mortality of CVD have continued to rise, and overtaken all other public health issues in terms of importance^4^. The impact of CVD on public health is increasingly aggravated. According to an updated report 2021 from China’s National Center for Cardiovascular Disease, in 2019, CVD remained the leading cause of death in urban and rural areas, and 330 million patients with CVD were estimated in China. The proportion of deaths from CVD among the total causes of death in rural and urban areas was 46.74$\%$ and 44.26$\%$, respectively. According to the report from China Health Statistics Yearbook 2020, in 2019, the mortality for CVD in rural was higher than that of in urban, and the mortality of males was higher than that of females.

Most of the previous studies have mainly focused on the impacts of climate change on public health^5–8^. For example, climate change can contribute to morbidity and mortality of CVD^9^. Some previous studies have shown that changes in the ambient temperature may cause hot and cold stress-related illness and contribute to CVD mortality by increasing blood pressure, blood viscosity, and heart rate^10–17^. Cold exposure, heatwaves, and diurnal temperature differences contribute to the risk of CVD^18^. The evidence of the previous studies on a relationship between CVD and the ambient temperature is most from developed countries (regions) or big cities^8,15,16,19–21^ but little from low- and middle-income countries (regions) where CVD is more serious^22,23^. Western China has a harsh climate and a higher risk of CVD, in addition, the short-term impact of the ambient temperature on CVD is different for different regions and populations due to potentially affected by climatic conditions, socioeconomic, demographic structure and living habits, etc.^22,23^.

Diurnal temperature range (DTR), an influential meteorological indicator of global climate change, is defined as the difference between the daily maximum and minimum air temperatures^15,24^. Compared with the average temperature of a single indicator, DTR can better characterize the magnitude of daily ambient temperature changes^24–26^. It has been reported that DTR has a significantly strong association with the increase in CVD morbidity and mortality^15,27^. However, the previous presented evidence suggesting on the relationship between CVD and DTR was limited, especially in northeast China where is cold weather in winter and poorer medical infrastructures. In addition, by analyzing the relationship between DTR and elevation, the existing research showed that the DTR or DTR change in northeast China is different from that in the other regions of China^28^. In Zhang et al.^28^, the study results showed that in northeast China, increasing elevation has a warm effect on DTR, leading to a significant increase of the DTR trend. Thus, we focused on the city of northeast China-Hulunbuir where is area with larger diurnal temperature difference and higher CVD mortality. To the best of our knowledge, it is the first study to explore how DTR affects CVD mortality in city of northeast China. In Inner Mongolia Autonomous Region, China, from 1990 to 2017, CVD mortality rate increased from 222.98/100,000 to 347.14/100,000, while CVD morbidity rate increased from 4142.47/100,000 to 8457.09/100,000^29^. CVD contributed more to mortality than the national average in Inner Mongolia Autonomous Region^30^. According to mortality data statistics between 2014 and 2020 from the 20 national cause-of-death monitoring stations, the region with the highest number of CVD mortality located primarily in the northeast and west-central areas of Inner Mongolia Autonomous Region, in which Hulunbuir had the highest CVD mortality cases, with recording a total of 21,067 CVD mortality (see Supplementary Fig. 1).

In 2013, three national cause-of-death monitoring stations were established in Hulunbuir, China. Our study aim was to explore the short-term effect of DTR on CVD mortality from 2014 to 2020 in Hulunbuir, China, using a distributed lag non-linear model (DLNM). We assessed both single-lag effect and cumulative effect, and estimated the overall and extreme effects of DTR on CVD mortality. The research evidence will be beneficial in assessing the possible short-term impact of future climate change on the health of specific groups, offering some suggestions for local public health policy formulation, health care resource allocation, and response to extreme climate disturbance.

## Methods

### Study area and data

**Figure 1:**
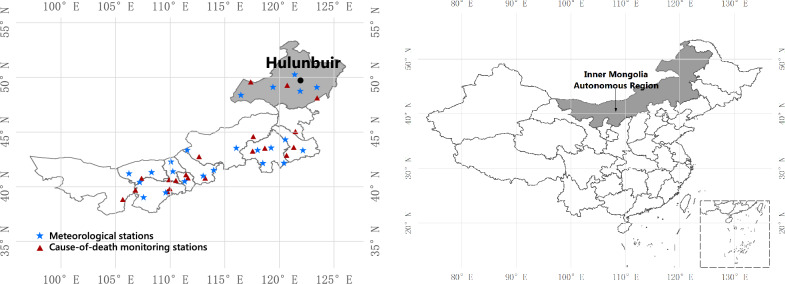
Geographical location of Hulunbuir, China: Hulunbuir city, Inner Mongolia Autonomous Region, China (left); China (right).

Hulunbuir, China ($46^{\circ } \;10^{\prime }$–$53^{\circ } \;26^{\prime }$ N,$115^{\circ } \;31^{\prime }$–$126^{\circ } \;04^{\prime }$ E), with a total area of 253,000 km$^{2}$ and a population of about 223,6300 in 2018 (National Bureau of Statistics of China), is located in northeast China, bordering Heilongjiang Province to the east, Mongolia to the west, and Russia to the north (Fig. 1). From east to west, climate changes from a cold-temperate continental monsoon climate to a mid-temperate continental climate with high winds and little precipitation in spring, brief mild summers, sharp temperature drops in fall, and long cold winters. The annual average temperature is between $-2.2\;^{\circ }$C and $2.4\;^{\circ }$C; and the difference in daily temperature is large. Due to the effect of DTR on CVD mortality may vary between warm season and cold season, we divided annual into two seasons with warm and cold seasons, in which spring and summer were regarded as warm season (from April to September, average DTR > annual average DTR), and fall and winter were regarded as cold season (from October to March of the next year, average DTR < annual average DTR)^31–33^.

Daily number of CVD mortality from January 1, 2014 to December 31, 2020 were collected by the Inner Mongolia Center for Disease Control and Prevention from the 3 national cause-of-death monitoring stations in Hulunbuir. Daily counts of CVD mortality are the total from the 3 monitoring stations. Figure 1 describes a district map of Hulunbuir, showing the locations of the 20 cause-of-death monitoring stations in Inner Mongolia Autonomous Region. The personal privacy information was not involved in the data set. The data set includes age, gender, diagnosis codes, and current residence. The causes of mortality were coded according to the 10${\text{ th }}$ edition of the International Classification of Diseases (ICD-10) with the following codes: chronic rheumatic heart disease with hypertension (ICD-10: I05–I15), ischemic heart disease with pulmonary heart disease, pulmonary circulation disease (ICD-10: I20–I28), and other types of heart disease (ICD-10: I30–I52). In addition, the CVD mortality data was divided into several subgroups by gender (male, female), age (< 65, $\ge$ 65 years), and season (cold, warm).

Daily meteorological data from January 1, 2014 to December 31, 2020 were obtained from the China Meteorological Science Data Sharing Service (https://data.cma.cn/) from the 23 meteorological stations in Inner Mongolia Autonomous Region, including daily minimum temperature ($^{\circ }$C), daily maximum temperature ($^{\circ }$C), relative humidity (%), sunshine duration (h), and average wind speed (m/s). Here, we selected 5 meteorological stations to investigate the climate change in Hulunbuir, China from 2014 to 2020. Figure 1 shows the distribution of the 5 meteorological stations in Hulunbuir. The average daily meteorological data from the 5 monitoring stations were converted into the daily meteorological data in Hulunbuir.

### DLNM model and parameter estimation

As pointed out by many previous studies, the association between DTR and CVD is non-linear and delayed in time^19,20,33,34^. Thus, a distributed lag non-linear model (DLNM) based on a quasi-Poisson regression model was applied to assessing the short-term effects of DTR on daily CVD mortality, while controlling for relative humidity (RH), average wind speed (AW), sunshine duration (SSD), day of the week, holiday, and the long-term trends of daily CVD mortality. In addition, we chose the final model in the analysis based on the Akaike information criteria (AIC) value (see Table 1) as follows:

1 $$\begin{aligned} \begin{array}{lll} Y_t&{}\sim &{}\text{ quasi-Poisson }(\mu _t)\\ \log (\mu _t)&{}=&{}\alpha +\beta \text{ DTR}_{t,l} +ns(\text{ Weather },df)+ns(\text{ Time },df)+\text{ Dow}_t+\text{ Holiday}_t, \end{array} \end{aligned}$$

where the subscript *t* denotes the day of the observation, $Y_t$ and $\mu _t$ denote the observed number and the expected number of daily CVD mortality on day *t*, respectively; $\alpha$ is the intercept term; $\text{ DTR}_{t,l}$ is the cross basis matrix obtained by applying DLNM to DTR over a lag of 0 (the current day) to *l* (the maximum lag days) days; $\beta$ is the vector of coefficients for $\text{ DTR}_{t,l}$; *ns*() indicates the natural cubic spline function, *df* is the degree of freedom; $\text{ Time }$ is adopted to control for long-term and seasonal trends; $\text{ Weather }$ represents meteorological factor taking into account relative humidity (RH), average wind speed (AW), and sunshine duration (SSD); $\text{ Dow}_t$ denotes the day of the week on day *t*, and $\text{ Holiday}_t$ is a category variable that the value is “1” if day *t* is a public holiday, otherwise it is “0”.

**Table 1:** AIC values for the relationship between average temperature and CVD mortality by DLNM type (The basic model only controlled long-term and seasonal trends, day of the week, holiday).

Model	AIC
Basic model	13359.01
Model 1 (basic model + average temperature)	13562.75
Model 2 (basic model + maximum temperature)	13559.62
Model 3 (basic model + minimum temperature)	13554.61

In this study, using a cubic spline function, we took the degree of freedom (*df*) as 6 per year for $\text{ Time }$ to eliminate long-term trend and seasonality. On the current day, we used 3 degrees of freedom (*df*) natural cubic spline to control the effects of RH, AW, and SSD^20,33,34^. The minimum value of AIC for quasi-Poisson models was applied for choosing the maximum lag days, *df*s for DTR and lags^19,20,34^. Finally, a maximum lag of 21 days was used to assess the delayed effect of DTR on CVD mortality. The cubic spline with both 4 *df* was used to model the non-linear exposure-response and lag-response associations between daily DTR and CVD mortality. The relative risk (RR) with a $95\%$ confidence interval (CI) was applied for evaluating the relationship between DTR and CVD mortality.

In the first stage, we analyzed the whole effect of DTR on CVD mortality along the 21 lag days by plotting the three-dimensional figure, in which the median DTR (11.20 $^{\circ }$C, $50{\text{ th }}$ percentile) was chosen as the reference value of DTR variable for calculating the RR values. In order to more specifically assess the characteristics of the DTR–CVD relationship, we estimated the RR values of CVD mortality by DTR at specific lags (0, 3, 14, and 21) and by lag at specific values of DTR (4.06 $^{\circ }$C, 5.90 $^{\circ }$C, 18.00 $^{\circ }$C, and 20.84 $^{\circ }$C), corresponding to $1{\text{ st }}$, $5{\text{ th }}$, $95{\text{ th }}$, and $99{\text{ th }}$ percentiles of DTR distribution (termed as extremely low, moderately low, moderately high, and extremely high DTR, respectively). In the second stage, stratified analyses by gender, age, and season were conducted to estimate the cumulative effects of DTR. We divided daily CVD mortality data into subgroups according to gender (female vs. male), age (< 65 years old vs. $\ge$ 65 years old), and season (warm season: April to September vs. cold season: October to March in next year). Furthermore, we analyzed the short-term effect of extremely high DTR on CVD mortality in each of subgroups, respectively.

Sensitivity analyses were also performed by reducing the controlled variables, altering *df* (7–9 per year) for time, changing *df* (4–6) for relative humidity, sunshine duration, and average wind speed. In addition, the maximum lag periods were adjusted from 14 days to 27, and 31 lags for DTR.

## Results

Table 2 lists the descriptive statistics of daily CVD mortality and meteorological factors in Hulunbuir, China, from 2014 to 2020. Over seven years, a total of 21,067 CVD mortality cases were recorded. The average number of daily deaths in Hulunbuir from 2014 to 2020 was 8.24 during the full year, 8.65 in the cold season and 7.83 in the warm season. The average daily deaths for the female, male, adults (age < 65 years), and old people (age $\ge$ 65 years) were 3.44, 4.80, 2.26, and 8.98, respectively. During the study period, the minimum, maximum, and average value of DTR were 2.10, 24.90, and 11.52, respectively. The mean DTR was 10.46 for the cold season and 12.58 for the warm season. The daily average values for air temperature, relative humidity, sunshine duration, and average wind speed were $11.52~ (\pm 17.03)$ $^{\circ }$C, 61.35 $(\pm 15.73)$ $\%$, 7.731 $(\pm 3.63)$h, and 4.439 $(\pm 1.89)$m/s, respectively.

**Table 2:** Descriptive statistics of daily CVD mortality cases and meteorological factors in Hulunbuir, China from 2014 to 2020.

Variables	Sum	Mean ± SD	Min	P25	P50	P75	Max
CVD cases							
Full year	21,067	8.24 ± 3.14	0	6	8	10	23
Cold season (Oct.–Mar.)	11,039	8.65 ± 3.06	0	6	8	11	23
Warm season (Apr.–Sep.)	10,028	7.83 ± 3.18	0	6	8	10	19
Female	8802	3.44 ± 1.91	0	2	3	5	11
Male	12,265	4.80 ± 2.36	0	3	5	6	14
Aged < 65 years	5770	2.26 ± 1.54	0	1	2	3	10
Aged $\ge$ 65 years	15,297	5.98 ± 2.64	0	4	6	8	20
Temperature ($^{\circ }$C)							
Full year	–	0.09 ± 17.03	− 38.80	− 16.60	2.90	15.60	29.50
Cold season (Oct.–Mar.)	–	− 14.30 ± 10.82	− 38.80	− 22.90	− 16.65	− 5.30	15.70
Warm season (Apr.–Sep.)	–	14.41 ± 7.15	− 11.30	9.80	15.60	19.60	29.50
Relative humidity (%)							
Full year	–	61.35 ± 15.73	14.00	51.00	65.00	73.00	94.00
Cold season (Oct.–Mar.)	–	66.90 ± 11.12	25.00	62.00	70.00	75.00	93.00
Warm season (Apr.–Sep.)	–	55.83 ± 17.60	14.00	43.00	56.00	69.00	94.00
Sunshine duration (h)							
Full year	–	7.73 ± 3.63	0.00	5.60	8.00	10.30	15.60
Cold season (Oct.–Mar.)	–	6.67 ± 2.80	0.00	5.40	7.10	8.70	12.60
Warm season (Apr.–Sep.)	–	8.79 ± 4.03	0.00	6.00	10.00	12.00	15.60
Average wind speed (m/s)							
Full year	–	4.44 ± 1.89	0.90	3.00	4.10	5.50	15.40
Cold season (Oct.–Mar.)	–	4.25 ± 1.68	1.10	3.00	4.00	5.30	11.30
Warm season (Apr.–Sep.)	–	4.62 ± 2.06	0.90	3.10	4.20	5.70	15.40
DTR ($^{\circ }$ C)							
Full year	–	11.52 ± 3.81	2.10	8.70	11.20	14.10	24.90
Cold season (Oct.–Mar.)	–	10.46 ± 3.31	2.10	8.10	10.00	12.50	24.20
Warm season (Apr.–Sep.)	–	12.58 ± 3.98	2.10	9.70	12.60	15.40	24.90

**Figure 2:**
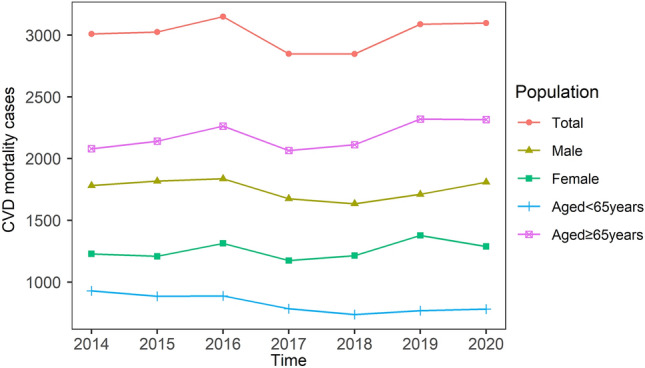
Time trends of annual CVD mortality in Hulunbuir, China, from 2014 to 2020.

**Figure 3:**
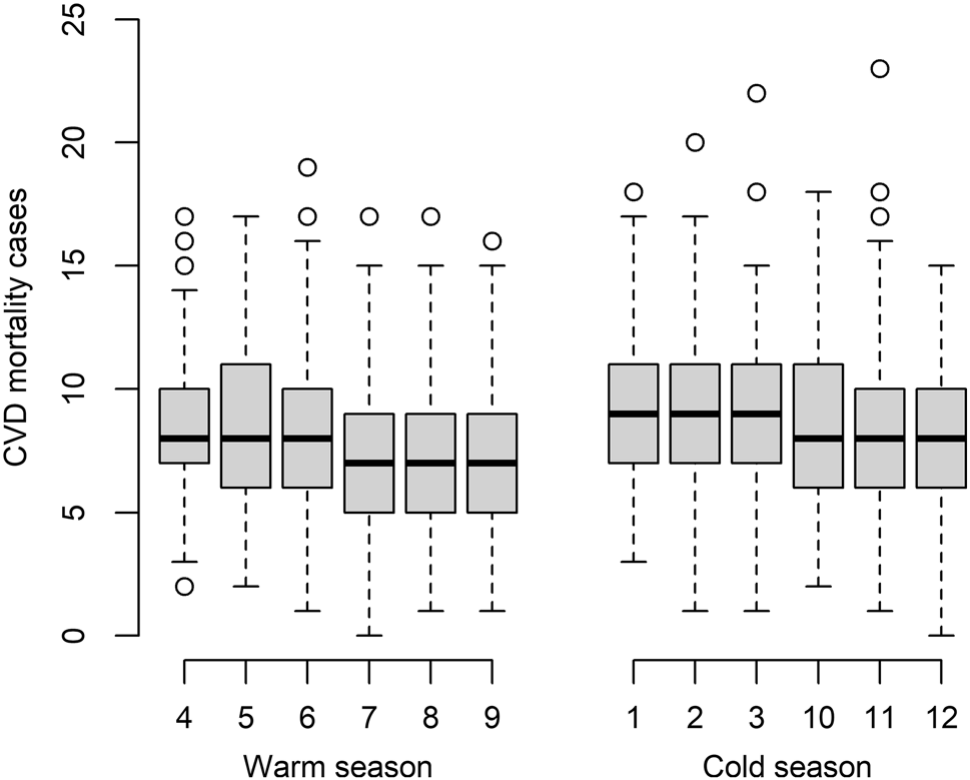
Seasonal and monthly distribution of daily CVD mortality cases in Hulunbuir, China, from 2014 to 2020.

The time series distribution of daily CVD mortality for total and different subgroups in Hulunbuir, China, from 2014 to 2020, is depicted in Fig. 2. Figure 2 shows that the number of CVD mortality climbed somewhat from 2014 to 2016, peaked in 2016, dropped quickly to a minimum in 2018, and then progressively increases from 2018 to 2020. The seasonal (warm and cold seasons) and monthly distribution of daily CVD mortality is shown in Fig. 3. There were relatively higher CVD mortality cases in cold season and lower CVD mortality cases in warm season. The number of daily CVD mortality was the most in January–March and the fewest in July–August.

Table 3 shows the Spearman’s correlation between DTR and other meteorological variables. DTR was negatively correlated with relative humidity, while DTR was positively correlated with both sunshine duration and temperature ($p<0.05$). Besides, DTR was slightly correlated with average wind speed, but the result was not statistically significant ($p>0.05$).

**Table 3:** Spearman’s correlation coefficients of each pair of the climate variables. ($$**p<0.01$$).

	DTR	Temperature	Relative humidity	Sunshine duration	Average wind speed
DTR	1.000	0.327**	− 0.602**	0.618**	− 0.011
Temperature	–	1.000	− 0.357**	0.370**	0.097**
Relative humidity	–	–	1.000	− 0.587**	− 0.262**
Sunshine duration	–	–	–	1.000	− 0.060**
Average wind speed	–	–	–	–	1.000

**Figure 4:**
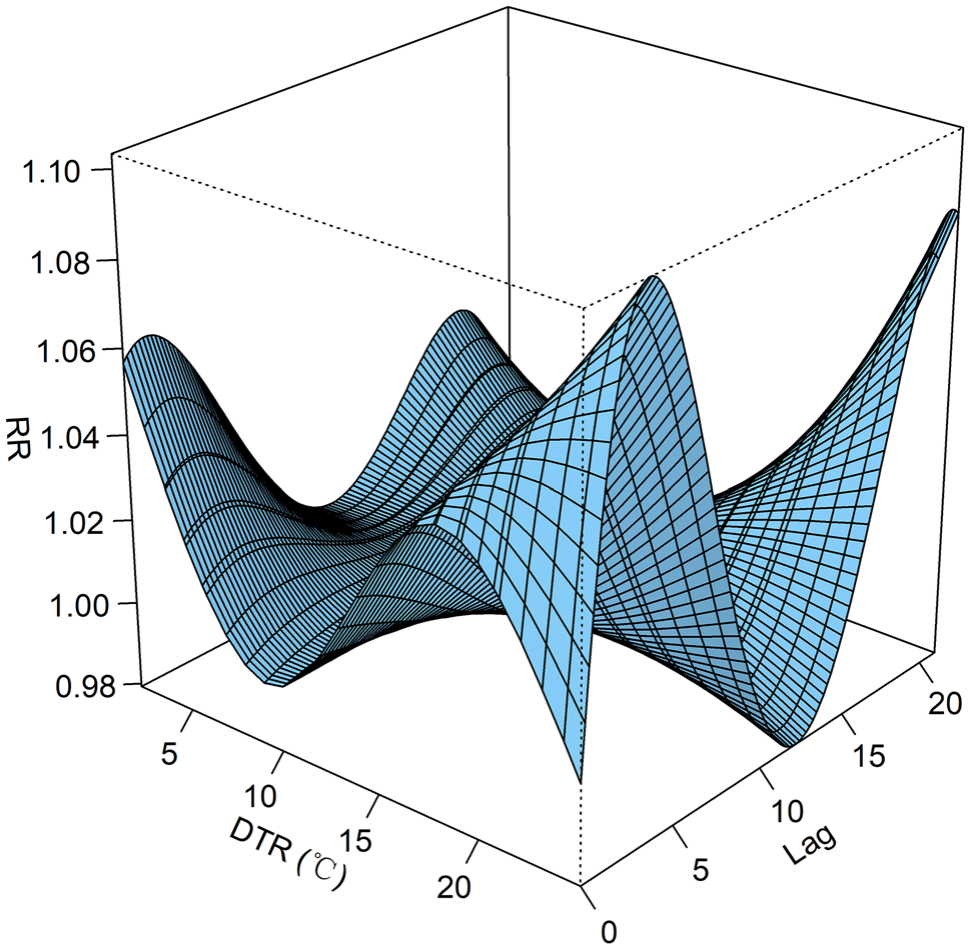
3-D graph of the relative risk (RR) of CVD mortality with DTR and lag (lag 0 to lag 21 days) using 11.20 $^{\circ }$C DTR (50${\text{ th }}$ percentile) as the reference value.

**Figure 5:**
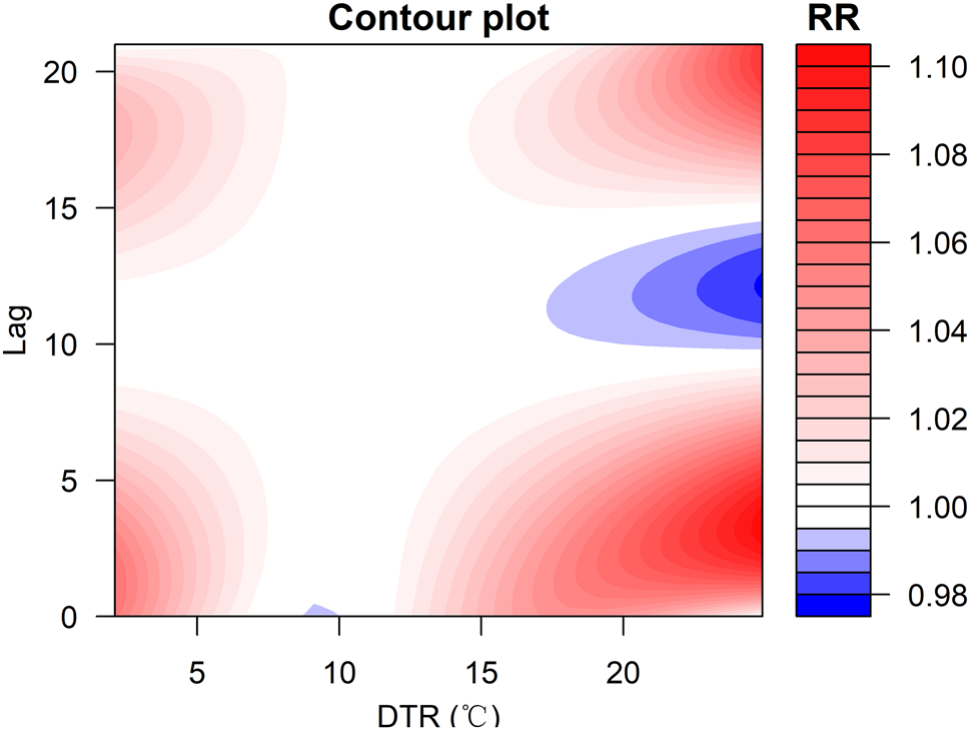
Contour of cumulative effects on CVD mortality, with reference at 11.20 $^\circ$C (50${\text{ th }}$ percentile).

The three-dimensional exposure-response surface of DTR on CVD mortality along lag days is shown in Fig. 4. The contour of the RR along DTR and lags on CVD mortality cases is shown in Fig. 5. Generally, the association between DTR and CVD mortality was non-linear. The estimated effects of lower and higher DTR on CVD mortality appeared immediately and persisted at least 3 days, with the highest mortality risks at 3 lags and 20 lags for higher DTR compared to DTR referenced at 11.20 $^{\circ }$C (50${\text{ th }}$ percentile). The trends of the RR values of CVD mortality were “M-shaped” and “N-shaped” along lag days under lower DTR and higher DTR, respectively.

**Figure 6:**
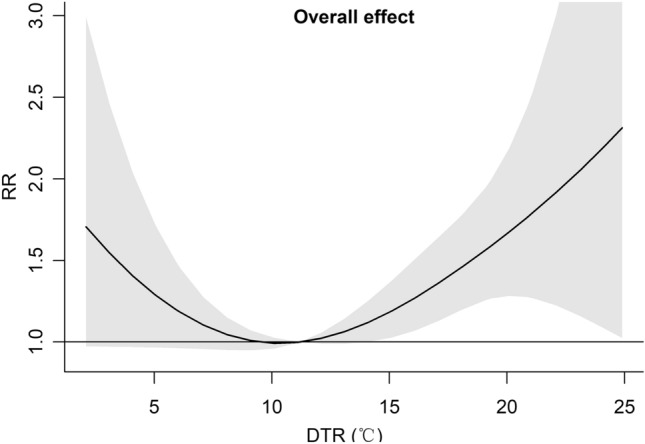
The overall relative risk (RR) of causing CVD mortality. Reference at 11.20 $^{\circ }$C DTR (50${\text{ th }}$ percentile). The $95\%$ CIs are represented by the shaded parts.

As shown in Fig. 6, the cumulative overall RR (95$\%$ CI) of causing CVD mortality along DTR in Hulunbuir, China, from 2014 to 2020 was “U-shaped”. The maximum RR value was 2.313 (95$\%$ CI 1.025–5.220) and occurred at a DTR of 24.90 $^{\circ }$C with the reference temperature 11.20 $^{\circ }$C. Compared to extremely low DTR, the RR values of CVD mortality increased sharply for extremely high DTR.

**Figure 7:**
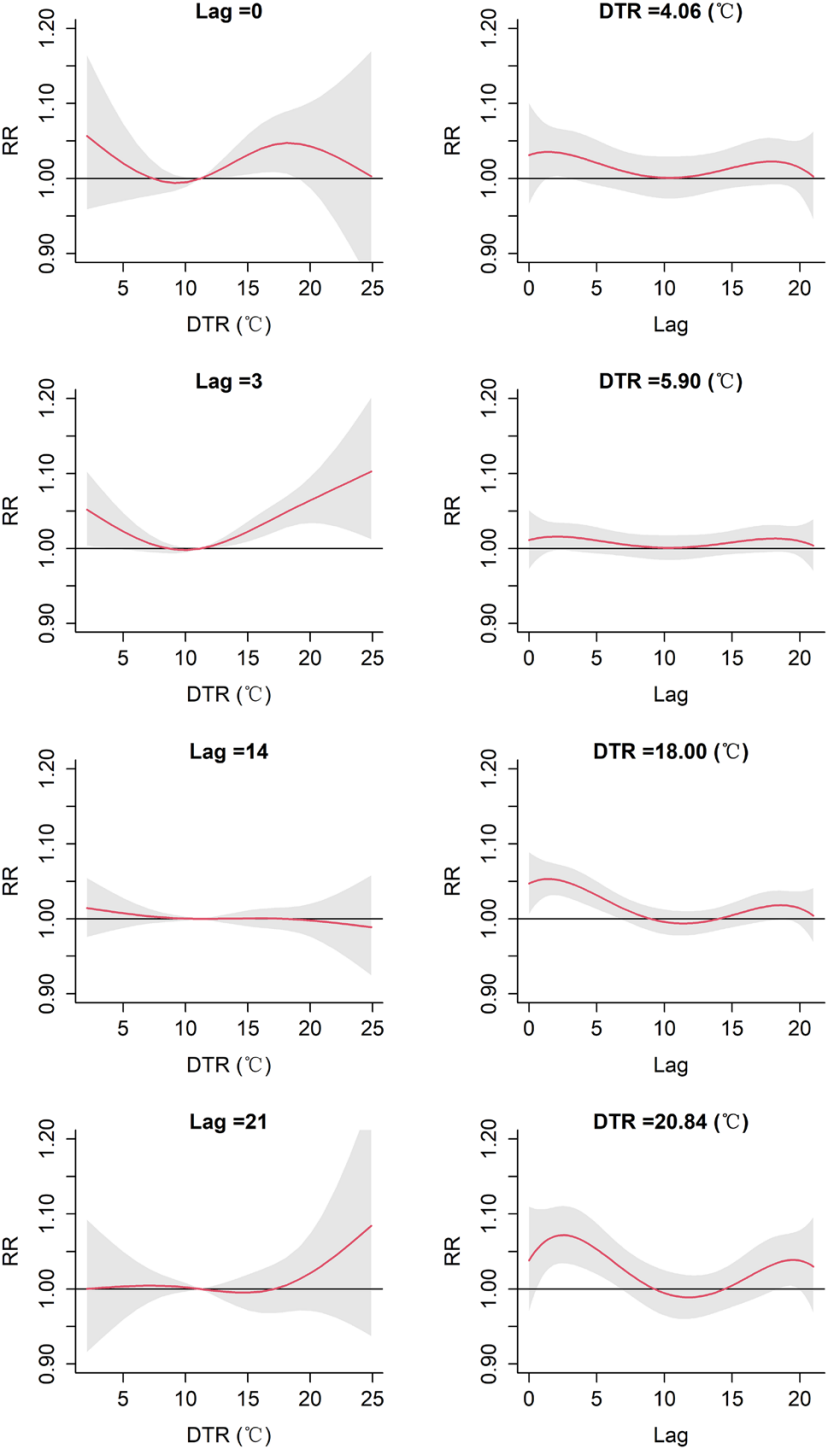
The relative risk (RR) of CVD mortality. Plot of RR by DTR at specific lags (left), RR by lag at 1${\text{ st }}$, 5${\text{ th }}$, 95${\text{ th }}$ and 99${\text{ th }}$ percentiles of DTR distribution (right). Reference at 11.20 $^{\circ }$C (50${\text{ th }}$ percentile).

Figure 7 shows the RRs ($95\%$ CI) of CVD mortality at the specified lag days (0, 3, 14, 21) and DTR (4.06 $^{\circ }$C, 5.90 $^{\circ }$C, 18.00 $^{\circ }$C, 20.84 $^{\circ }$C), where the DTR corresponds to extremely low DTR ($1{\text{ st }}$), moderately low DTR ($5{\text{ th }}$), moderately high DTR ($95{\text{ th }}$), and extremely high DTR ($99{\text{ th }}$), respectively. The cumulative effects of DTR on CVD mortality at different lags were depicted in the left of Fig. 7. For different levels of DTR, the cumulative effects of lag days on CVD mortality were shown in the right of Fig. 7. The results show that extremely low DTR (4.06 $^{\circ }$C) had a significant effect on CVD mortality at 3 lag days; moderately high DTR (18.00 $^{\circ }$C) and extremely high DTR (20.84 $^{\circ }$C) had significant effects on CVD mortality and the adverse impacts will persist at least 5 days.

Table 4 illustrates the RR (95$\%$ CI) of extremely high DTR on CVD mortality with DTR referenced at 11.20 $^{\circ }$C at different lag days (lag 0, lag 0–3, lag 0–7, lag 0–14, and lag 0–21) for female and male gender, age < 65 and age $\ge$ 65, respectively in Hulunbuir, China, from 2014 to 2020. According to the RR values displayed in Table 4, comparison among female and male groups revealed that males were more vulnerable to extremely high DTR compared with females. Comparison among the age < 65 and the age $\ge$ 65 groups revealed that extremely high DTR had a more adverse effect on CVD mortality for the age $\ge$ 65 group when lag period was ranged from 0 to 3 days, while the age < 65 group more vulnerable to extremely high DTR for the rest lag periods.

**Table 4:** Cumulative effects of extremely high DTR (99${\text{ th }}$ percentile) on CVD mortality by different population characteristics along the lag days in full year (extremely high DTR: 20.84 $^{\circ }$C. The bolding values represented statistically significant results. $p<0.05$).

Group	Lag 0	Lag 0–3	Lag 0–7	Lag 0–14	Lag 0–21
Female	$1.001\ (0.907, 1.106)$	$1.146\ (0.918, 1.430)$	$\mathbf{1.413\ (1.034, 1.930)}$	$1.448\ (0.964, 2.175)$	$1.298\ (0.808, 2.084)$
Male	$1.067\ (0.978, 1.161)$	$\mathbf{1.355\ (1.116, 1.644)}$	$\mathbf{1.585\ (1.203, 2.087)}$	$\mathbf{1.503\ (1.047, 2.158)}$	$\mathbf{2.210\ (1.450, 3.367)}$
Aged $<65$ years	$\mathbf{0.877\ (0.771, 0.998)}$	$0.996\ (0.749, 1.323)$	$\mathbf{1.521\ (1.028, 2.250)}$	$\mathbf{1.886\ (1.134, 3.137)}$	$\mathbf{2.867\ (1.583, 5.192)}$
Aged $\ge 65$ years	$\mathbf{1.099\ (1.019, 1.185)}$	$\mathbf{1.364\ (1.149, 1.619)}$	$\mathbf{1.492\ (1.167, 1.907)}$	$1.332\ (0.966, 1.838)$	$\mathbf{1.459\ (1.004, 2.121)}$

Table 5 displays the RR (95$\%$ CI) of extremely high DTR (i.e., full year: 20.84 $^{\circ }$C, warm season: 21.80 $^{\circ }$C, and cold season: 18.85 $^{\circ }$C) on CVD mortality at different lag days in full year and two seasons. In full year, adverse effects had increased along lag days. Comparison among warm season and cold season showed that extremely high DTR had a more averse effect on CVD mortality in cold season.

**Table 5:** Cumulative effects of extremely high DTR on CVD mortality along the lag days in full years and two seasons (warm and cold) (extremely high DTR: full year: 20.84 $^{\circ }$C, warm season: 21.80 $^{\circ }$C, cold season: 18.85 $^{\circ }$C. The bolding values represented statistically significant results. $p<0.05$).

Season	Lag 0	Lag 0–3	Lag 0–7	Lag 0–14	Lag 0–21
Full year	$1.038\ (0.971, 1.110)$	$\mathbf{1.263\ (1.087, 1.466)}$	$\mathbf{1.509\ (1.221, 1.865)}$	$\mathbf{1.474\ (1.118, 1.945)}$	$\mathbf{1.762\ (1.276, 2.432)}$
Warm season	$1.001\ (0.906, 1.105)$	$1.102\ (0.864, 1.405)$	$1.165\ (0.796, 1.704)$	$1.018\ (0.572, 1.814)$	$1.255\ (0.595, 2.644)$
Cold season	$1.017\ (0.934, 1.108)$	$\mathbf{1.262\ (1.041, 1.530)}$	$\mathbf{1.552\ (1.162, 2.073)}$	$1.406\ (0.888, 2.225)$	$1.510\ (0.808, 2.824)$

## Sensitivity analysis

Overall, sensitivity analyses indicated that our study results were robust to the reduction of controlled variables (Supplementary Fig. 2), change of *df* (7, 8, and 9) for time per year (Supplementary Fig. 3), and the change of *df* (4, 5, and 6) for relative humidity, sunshine duration, and average wind speed (Supplementary Fig. 4). Moreover, we also used different maximum lag days for DTR to fit the model, the estimated results did not substantially change (Supplementary Fig. 5). Furthermore, when excluding data from 2020 in the DLNM, the result was still similar to the original estimates (Supplementary Fig. 6).

## Discussion

This article investigates the short-term effects of DTR on CVD mortality in Hulunbuir, China. Here, a quasi-Poisson generalized linear regression combined with a distribution lag non-linear model (DLNM) was used to explore the exposure-response relationship and displayed impacts between DTR and CVD mortality. To the best of our knowledge, it was the first study in Hulunbuir located in northeast China. The study results show that the exposure-response association between DTR and CVD mortality was non-linear with “U-shaped”. The non-linear association was consistent with most previous studies^20,23,33,35,36^. In fact, some presented study results have shown that DTR was correlate with CVD and the relationship between DTR and CVD was non-linear^15,20,21,23,33^. Both extremely low and extremely high DTR had adverse short-term effects on CVD mortality. The maximally adverse effect occurred at extremely high DTR, and extremely high DTR had a significant adverse effect on CVD mortality. Compared with the female and the age < 65 groups, the male and age $\ge$ 65 groups were more likely to be affected by extremely high DTR. Furthermore, for season factors, our results indicated that the effects of extremely high DTR on CVD in cold season had more adverse than in warm season.

High DTR had a more detrimental impact on CVD. In the analysis of specific DTR, we found that extremely high DTR had a more significant short-term impact on CVD mortality, which was consistent with the findings of some earlier studies^21,23,36^. The extremely high DTR means big fluctuations in temperature over day that may increase systolic and pulse pressure and decrease diastolic blood pressure, and the large increase in blood pressure may enhance the RR of CVD mortality to some extent^37^. However, there were also other findings. For example, in Qingyang, Gansu, China, both low and high DTR had a detrimental impact on CVD hospitalization, and low DTR had a higher impact compared with high DTR^23^. This may be because residents working in suburban areas can arrange their working hours more flexibly and take prompt countermeasures according to difference in outdoor temperature, which can avoid the health effects of large changes in temperature. Therefore, the association between DTR and CVD may be affected by the study area and the production and lifestyle of residents.

Many studies have shown that factors such as gender and age could modify the short-term effects of DTR on CVD mortality^20,21,33,38^. The cumulative effects of extremely high DTR on CVD mortality by different population characteristics (gender, age) were estimated in our study. It found that males and older people (age $\ge$ 65 years) were more vulnerable to extremely high DTR compared with females and younger people (age < 65 years) for CVD mortality. It was confirmed that extremely high DTR has a greater adverse short-term effect on CVD mortality in males and older people (aged $\ge$ 65 years) than in females and young people (aged < 65 years)^20,33^. However, some previous studies showed different results^15^. For example, the study results in Shanghai, China, found no statistically significant difference between male and female in the impact of DTR on CVD mortality^15^. This may be because the effect of temperature on the population was limited by the different study areas and study populations^39^. This might occur because the male group would smoke and drink alcohol more frequently and be more exposed to the outdoors compared with the female group, older people have a relatively poorly functioning thermoregulatory system and may also have some underlying medical conditions, which do not adapt well to significant changes in temperature as a susceptible population^5^.

The previous studies have shown that season as modifying factor influenced the association between DTR and CVD mortality^15,16,20,35,38^. According to the climatic characteristics of Hulunbuir, we divided the full year into two seasons (cold and warm) for the study, and found that extremely high DTR was associated with greater adverse short-term impact in cold season compared with warm season. Therefore, cold season strengthened the association between extremely high DTR and CVD mortality, which was consistent with the results of the previous studies^21,35,38^. Possible reasons for the increased relative risks of CVD mortality include that for people exposed to cold weather, cold sensory receptors on the skin and certain mucous membranes transmit changes in external ambient temperature to the sympathetic nervous system, which increases the release of catecholamines in the body, which in turn causes vasoconstriction, increases myocardial contractility, elevates heart rate, increases blood pressure, and increases platelets and blood viscosity, all of which are important physiological changes that are risk factors for triggering CVD or even causing CVD mortality^8,40,41^.

## Conclusion

This study has some strengths. Our study may be the first study to assess the non-linear effect and lag effect of DTR on CVD mortality in Hulunbuir, China. Moreover, we explored whether the factors of season, gender, and age associated with the effects of DTR on CVD mortality. Our study might provide some scientific basis for studying the prevention and treatment of regional CVD. Hulunbuir had a large variation in DTR (from 2.10 to 24.90 $^{\circ }$C, between 2014 to 2020) and a long cold period. Therefore, residents, especially males and older people (age $\ge$ 65 years), should pay attention to making timely countermeasures according to the ambient temperature. When DTR is high (about 20.84 $^{\circ }$C) and in a cold environment, people should promptly adjust outdoor working hours, and add clothes to mainly avoid the impact of large temperature changes on CVD mortality.

However, several limitations of this study should be noted. First, the study data were only obtained from Hulunbuir, China. Due to the differences in geographic position, climatic environment, and economic growth among various locations, the study results should be cautiously applied to other cities. Second, we used meteorological data measured from fixed meteorological monitoring stations as a proxy for individual exposure levels, so there were some unavoidable biases in the model estimation results.

In summary, the association of DTR with CVD mortality in Hulunbuir, China, was non-linear, and the study results reinforced the findings of some previous studies in which there was a non-linear association between DTR and CVD mortality. Specifically, extremely high DTR had a significant adverse short-term effect on CVD mortality. In addition, we found no significant effects of extremely high DTR on CVD mortality for different lag periods in warm season, and cold season strengthened the adverse effects. Males and older people (age $\ge$ 65 years) were more susceptible to extremely high DTR compared with the female and adult (age < 65 years) groups. As the first study conducted to assess the correlation between DTR and CVD mortality in Hulunbuir, China, our findings may provide some theoretical basis for local public health departments designing prevention and control policies to reduce the short-term impacts of extremely high DTR on vulnerable groups.

## Ethics declarations

This study does not involve human experiments, and uses CVD mortality dataset from the Inner Mongolia Center for Disease Control and Prevention. All methods were carried out in accordance with relevant guidelines and regulations. Ethical approval was obtained from the Ethics Committee of Affiliated Hospital of Inner Mongolia Medical University. Informed consent was obtained from all subjects legal guardian(s) involved in the study. The information collected in this study contained date of birth, gender, age, date of death, place of residence, and did not include patient’s name and ID number, and the personal information about patients were fully protected.

## Supplementary Information

Supplementary Information 1.

Supplementary Information 2.

## Data Availability

The data described during this study can be found in supplementary information files of this article. In this paper, R software (version 4.1.1, R Development Core Team 2021) was used to fit all models and statistical analyses. The DLNM analysis was conducted using the “dlnm” package.
