# Supplementary material for: Short-term impact of diurnal temperature range on cardiovascular diseases mortality in residents in northeast China
Source: Sci Rep. 2023 Jul 7;13:11037. doi: 10.1038/s41598-023-38129-2 (PMC10328923; doi:10.1038/s41598-023-38129-2)
Supplement: Supplementary file 1 — Supplementary Information 1. [file 41598_2023_38129_MOESM1_ESM.pdf]

Online Supplemental Material

Title: Short-term impact of diurnal temperature range on cardiovascular diseases mortality in residents in northeast China

Xuan Kai[1], Zhimin Hong[1,*], Yang Hong[2,+], Xiaolei Wang[3], and Chunyang Li[4]

[1] School of Sciences, Inner Mongolia University of Technology, Department of Mathematics, Hohhot, 010051, China

[2] School of Sciences, Inner Mongolia University of Technology, Department of Mechanics, Hohhot, 010051, China

[3] Affiliated Hospital of Inner Mongolia Medical University, Department of Ultrasound, Hohhot, 010050, China

[4] Affiliated Hospital of Inner Mongolia Medical University, Department of Neurology, Hohhot, 010050, China

[*]zhmhong@imut.edu.cn

[+]this author contributed equally to this work

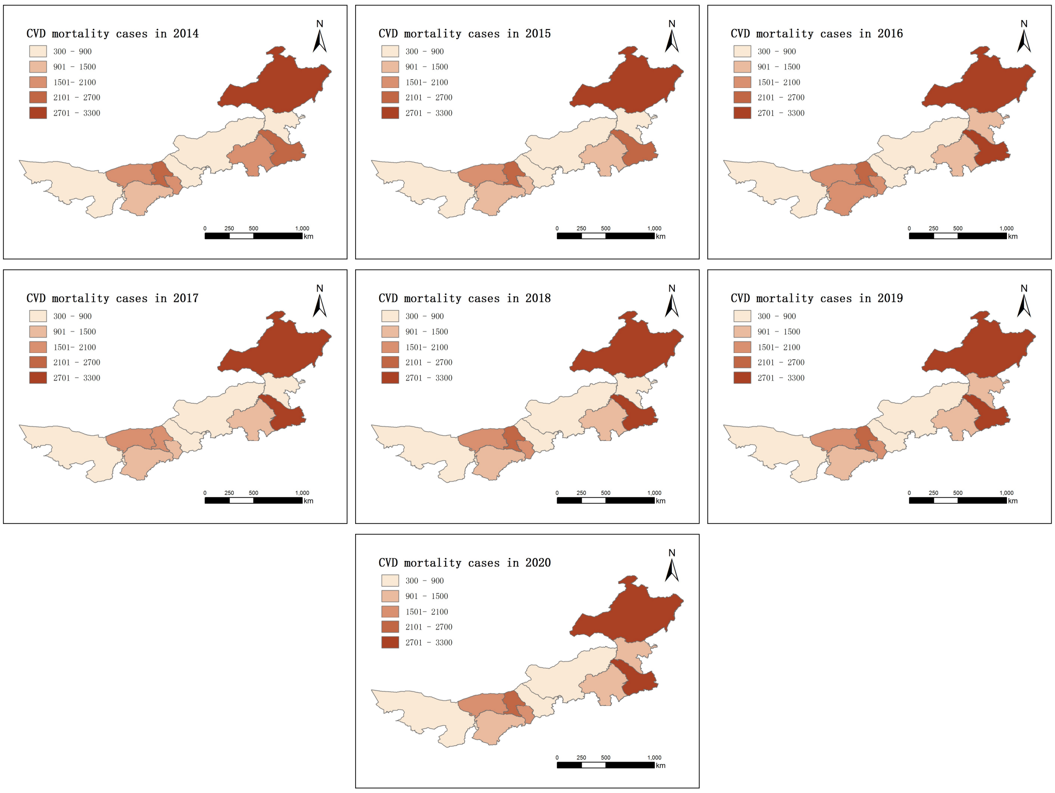

S.Fig. 1 Spatial distribution of cardiovascular disease (CVD) mortality cases based on the 20 national cause-of-death monitoring stations in Inner Mongolia Autonomous Region, China from 2014 to 2020.

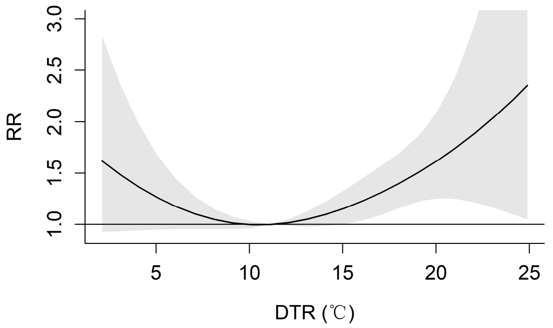

S.Fig. 2 Cumulative exposure-response relationship over a lag of 21 days with reduced model which taken Time trend, day of the week, public holiday and DTR into consideration only.

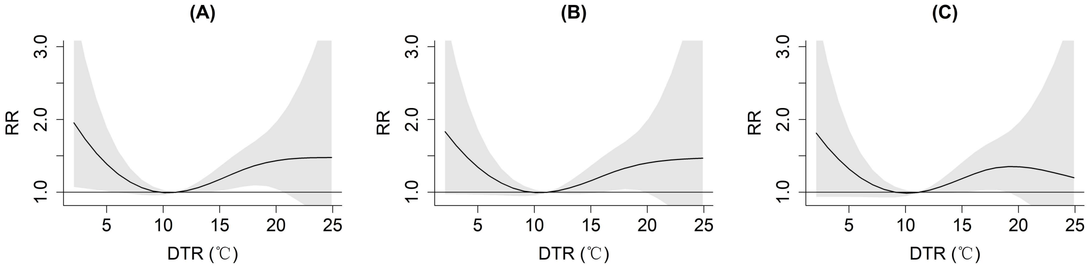

S.Fig. 3 Cumulative exposure-response relationship over a lag of 21 days, using (A) 7, (B) 8 and (C) 9 df per year for Time, respectively.

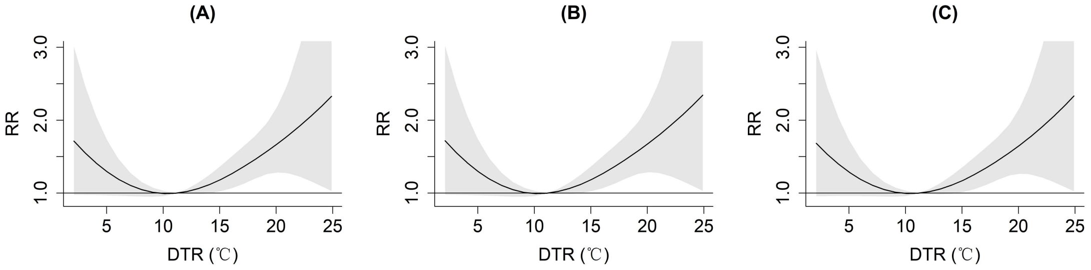

S.Fig. 4 Cumulative exposure-response relationship over a lag of 21 days, using (A) 4, (B) 5 and (C) 6 df for relative humidity, sunshine duration, and the average value of wind speed, respectively.

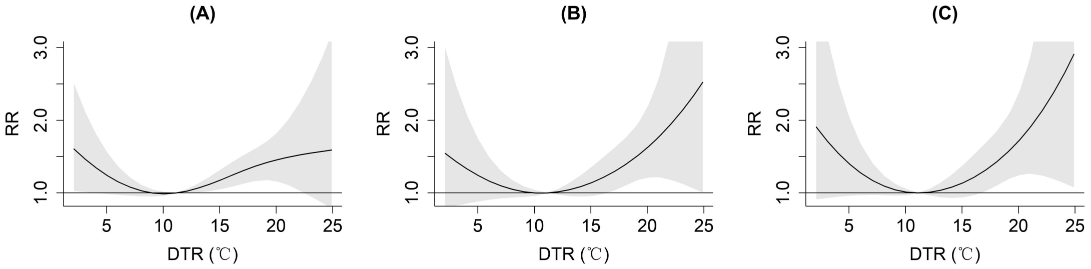

S.Fig. 5 Cumulative exposure-response relationship for different lag days, using (A) 14, (B) 27 and (C) 31 for max lag days, respectively.

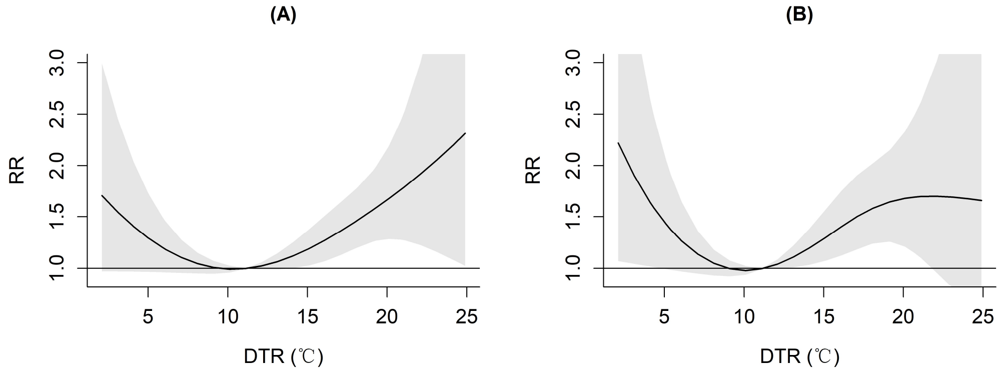

S.Fig. 6 Cumulative exposure-response relationship along 21 lag days. (A) during 2014 and 2020; (B) excluding data from 2020, respectively.
